# Inhibitory Effects of Cinnamaldehyde Derivatives on Biofilm Formation and Virulence Factors in *Vibrio* Species

**DOI:** 10.3390/pharmaceutics13122176

**Published:** 2021-12-17

**Authors:** Olajide Sunday Faleye, Ezhaveni Sathiyamoorthi, Jin-Hyung Lee, Jintae Lee

**Affiliations:** School of Chemical Engineering, Yeungnam University, 280 Daehak-Ro, Gyeongsan 38541, Korea; osfaleye@yu.ac.kr (O.S.F.); 22000138@ynu.ac.kr (E.S.)

**Keywords:** antibiofilm, antivirulence, cinnamaldehyde, cinnamaldehyde derivatives, *Vibrio parahaemolyticus*

## Abstract

*Vibrio parahaemolyticus* is considered one of the most relevant pathogenic marine bacteria with a range of virulence factors to establish food-related gastrointestinal infections in humans. Cinnamaldehyde (CNMA) and some of its derivatives have antimicrobial and antivirulence activities against several bacterial pathogens. This study examined the inhibitory effects of CNMA and its derivatives on biofilm formation and the virulence factors in *Vibrio* species, particularly *V. parahaemolyticus.* CNMA and ten of its derivatives were initially screened against *V. parahaemolyticus* biofilm formation, and their effects on the production of virulence factors and gene expression were studied. Among the CNMA derivatives tested, 4-nitrocinnamaldehyde, 4-chlorocinnamaldehyde, and 4-bromocinnamaldehyde displayed antibacterial and antivirulence activities, while the backbone CNMA had weak effects. The derivatives could prevent the adhesion of *V. parahaemolyticus* to surfaces by the dose-dependent inhibition of cell surface hydrophobicity, fimbriae production, and flagella-mediated swimming and swarming phenotypes. They also decreased the protease secretion required for virulence and indole production, which could act as an important signal molecule. The expression of QS and biofilm-related genes (*aphA*, *cpsA*, *luxS*, and o*paR*), virulence genes (*fliA*, *tdh*, and *vopS*), and membrane integrity genes (*fadL*, and *nusA*) were downregulated in *V. parahaemolyticus* by these three CNMA analogs. Interestingly, they eliminated *V. parahaemolyticus* and reduced the background flora from the squid surface. In addition, they exhibited similar antimicrobial and antibiofilm activities against *Vibrio harveyi*. This study identified CNMA derivatives as potential broad-spectrum antimicrobial agents to treat biofilm-mediated *Vibrio* infections and for surface disinfection in food processing facilities.

## 1. Introduction

*Vibrios* are cosmopolitan, motile, aerobic, or facultatively anaerobic bacteria found in the marine environment. While some species, such as *V. parahaemolyticus*, *V. cholerae*, and *V. vulnificus*, are considered major human pathogens, other species, including *V. mimicus*, *V. fluvialis*, *V. alginolyticus*, *V. cincinnatiensis*, *V. harveyi*, *V. furnissii*, *V. damsela*, *V. metschnikovii*, and *V. carchariae*, are not often detected from patients suffering from gastroenteritis. They are regarded as occasional human pathogens [1].

*V. parahaemolyticus* is considered a frequent foodborne pathogen compared to other *Vibrio* species and accounts for the majority of reported cases annually, with its attendant economic loss in aquaculture [2,3]. The species is a Gram-negative halophilic bacterium found in two typical morphologies: a rigid curve or straight. The bacterium is a non-spore-forming, indole-positive, rod-shaped inhabitant of estuarine waters, marine environments, and seafood, such as fish, shrimp, and shellfish [4,5]. It encodes a wide array of virulence factors, including biofilm formation, protease secretion, cell surface hydrophobicity, motility by either polar or lateral flagellum, hemolysin, specialized type III secretion systems (T3SS), type VI secretion systems (T6SS), and polysaccharides [4,6,7]. Furthermore, *AphA* and *OpaR* are the two master quorum-sensing (QS) regulators in *V. parahaemolyticus* [8] that individually or synergistically mediate multiple cellular activities, including virulence factor production [9,10].

Biofilms are characterized as multilayer protected microbial establishments that are resistant to an array of antibiotics. They pose major problems in the food industry because they present on suitable surfaces, including stainless steel, glass, rubber, and even seafood products that support bacterial adherence [11]. Several studies reported the ability of *V. parahaemolyticus* to attach to the surfaces of shrimp, crab, mussels, smoked salmon, and prawns [7,12,13,14]. Its ability to form biofilms on suspended particles, zooplankton, fish, and shellfish is related to its surface adherence properties [15]. Until now, prophylactic antibiotics were extensively used in mariculture. On the other hand, their overuse led to increased resistance rates among clinical and aquatic isolates of *V. parahaemolyticus*; therefore, novel approaches are needed to manage vibriosis [3,16,17]. Antivirulence therapy interfering with bacterial virulence factors is an important alternative because it induces less selective pressure than antibacterial agents for the development of bacterial resistance [6,18].

Essential oils from the cinnamon plant have wide-spectrum antimicrobial effects because of the high presence of cinnamaldehyde [19]. Cinnamaldehyde (CNMA) is a non-toxic synthetic flavoring agent with a range of powerful pharmacological functions that are generally recognized as safe [20]. Previous studies on its microbiological activities have documented marked antimicrobial efficacy against food-related pathogens [21,22]. CNMA and its derivatives inhibit planktonic cell growth, biofilm production, and help eradicate microbial persister cells [23]. For example, trans-cinnamaldehyde was reported to inhibit biofilm formation in clinical pathogens, such as *Escherichia coli*, uropathogenic *E. coli*, enterohemorrhagic *E. coli* O157: H7 [24,25,26], and *Pseudomonas aeruginosa* (PAO1) [27]. In addition, its derivatives, such as 2 and 4-nitrocinnamaldehyde and α-bromocinnamaldehyde, were found to be active against the biofilms of *Streptococcus pyogenes* [28] and *E. coli* MG 1655 [23], respectively, while 2-NitroCNMA interfered with biofilm formation and autoinducer-2-based QS of *V. anguillarum* LMG 4411, and *V. harveyi*, respectively [3]. Additionally, CNMA demonstrated antifungal properties against multispecies biofilms of *Candida* spp. and virulent toxin production in *Aspergillus flavus* [29,30].

CNMA and cinnamon extracts have been evaluated for their antimicrobial and antivirulence properties against some strains of *V. parahaemolyticus* [31,32,33]. On the other hand, the effects of its derivatives on the virulence factors of *V. parahaemolyticus* are unclear. Some CNMA derivatives have enhanced activity compared to their backbone and even commonly used antibiotics [34]. For example, 4-NitroCNMA was reported to be significantly more active than the parent CNMA against *V. anguillarum* LMG 4411 [3]. Therefore, this study evaluated the antibiofilm and antivirulence potentials of CNMA and ten (10) of its derivatives against *V. parahaemolyticus* and *V. harveyi* as well as investigated the possible mechanism of action using transcriptional analysis and morphological examination by scanning electron microscopy (SEM) to elucidate their effects on biofilms. The virulence factors including motility, cell surface hydrophobicity, fimbriae activity, protease and indole production were examined with the most active CNMA derivatives.

## 2. Materials and Methods

### 2.1. Strain, Chemicals, and Culture Materials

**Figure 1:**
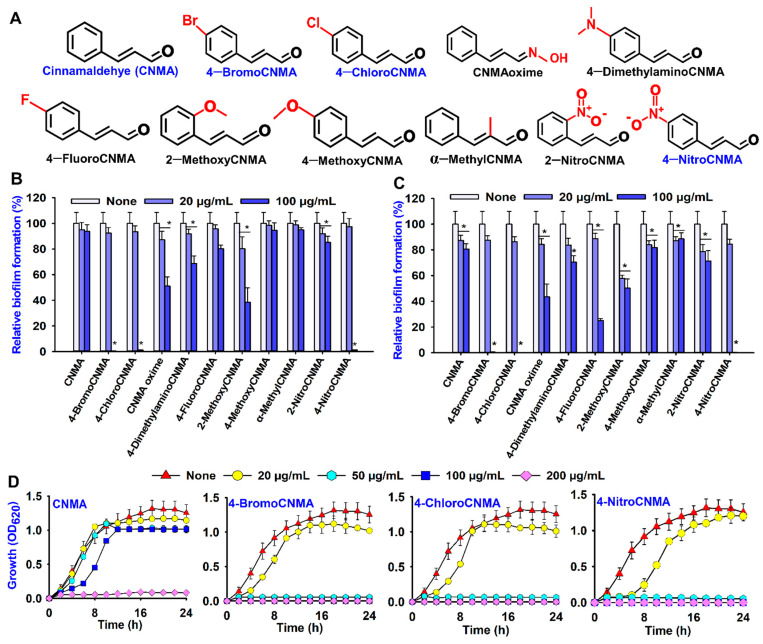
Structures and antibiofilm activities of cinnamaldehyde (CNMA) and its derivatives. All chemical structures (**A**), antibiofilm screening against *V. parahaemolyticus* (**B**), *V. harveyi* (**C**), growth curve of *V. parahaemolyticus* treated with different concentrations of CNMA and its selected derivatives (**D**). * denotes a significant difference at *p* < 0.05.

The bacterial strains used in this study were *V. parahaemolyticus* strain ATCC 17802 and *V. harveyi* ATCC 14126 (American Type Culture Collection, Manassas, VA, USA). Medium marine Luria-Bertani (mLB) containing 3% (wt./vol.) NaCl was purchased from Becton Dickinson (Franklin Lakes, NJ, USA). The test strains were transferred from a glycerol stock −80 °C to an mLB agar plate and incubated overnight at 30 °C. For the assays, a single colony from each plate was inoculated into 2 mL of an mLB broth and incubated overnight at 30 °C in a shaking incubator at 250 rpm. CNMA and ten of its derivatives and other chemicals were purchased from Sigma-Aldrich (St. Louis, MO, USA) and Combi-Blocks, Inc. (San Diego, CA, USA): cinnamaldehyde (CNMA), 4-bromocinnamaldehyde (4-BromoCNMA), 4-chlorocinnamaldehye (4-ChloroCNMA), cinnamaldehyde oxime (CNMA oxime), 4-dimethylaminocinnamaldehyde (4-DimethylaminoCNMA), 4-fluorocinnamaldehyde (4-FluoroCNMA), 2-methoxycinnamaldehyde (2-MethoxyCNMA), 4-methoxycinnamaldehyde (4-MethoxyCNMA), α-methylcinnamaldehyde (α-MethylCNMA), 2-nitrocinnamaldehyde (2-NitroCNMA), and 4-nitrocinnamaldehyde (4-NitroCNMA) (Figure 1A). The chemicals were solubilized in dimethyl sulfoxide (DMSO). Crystal violet was purchased from Sigma-Aldrich Co. (St. Louis, MO, USA). DMSO was also used as a negative control in all experiments at 0.1% (*v*/*v*) in media; this did not affect cell growth or the antibiofilm activity. The freshly frozen squids (*Todarodes pacificus*) used for the biotic surface assay were purchased from a local market (Gyeongsan, South Korea), covered, and quickly taken to the laboratory under ice for further processing.

### 2.2. Planktonic Cell Growth and Minimum Inhibitory Concentrations (MICs)

For the cell growth experiments, the *V. parahaemolyticus* and *V. harveyi* cells were inoculated into a 96-well plate mLB medium (1:100 dilution) treated with or without CNMA or its derivatives at different concentrations and incubated at 30 °C for 24 h. Growth was assessed spectrophotometrically at OD_620_ using a Multiskan EX microplate photometer [35]. The minimum inhibitory concentration (MIC) was determined using marine Luria-Bertani (mLB) containing 3% (wt./vol.) NaCl in 96-well polystyrene plates [32]. Briefly, overnight cultures of the test bacteria in 96-well plates were treated with CNMA and its derivatives at various concentrations (20, 50, 100, 200, 400 or 500 µg/mL) and incubated at 30 °C for 24 h. The MIC was defined as the lowest concentration to inhibit visible growth after incubation. The results are the averages of at least three independent cultures.

### 2.3. Crystal Violet Biofilm Inhibition Assay

This assay was carried out to analyze the inhibitory effects of CNMA and its derivatives on biofilm formation in *V. parahaemolyticus* and *V. harveyi*, as previously described with a slight modification [35,36]. Briefly, bacterial cells were inoculated into mLB at a dilution of 1:100 and incubated in the presence or absence of the test compounds at varying concentrations (20, 50, 100 or 200 µg/mL) for 24 h at 30 °C without shaking. The edge effects were avoided by adding the same amount of mLB to the peripheral wells (300 μL) of the 96-well plates used. Biofilm formation was quantified by removing the non-adherent cells by washing three times with sterile water. The biofilm was then stained with crystal violet 0.1% for 20 min. The excess dye was removed by washing, and the bound crystal violet was solubilized in 95% ethanol. The absorbance was measured at 570 nm using a Multiskan EX microplate photometer (Thermo Fisher Scientific, Waltham, MA, USA). The results are presented as the averages of measurements taken from three independent cultures with six replicates.

### 2.4. Biofilm Dispersal Assay

The eradicating effects of CNMA and its derivatives on preformed biofilms were investigated. The test bacterium was inoculated in a 96-well plate in the absence of CNMA and its derivatives for 24 h at 30 °C. After incubation, the broths were removed carefully by pipetting and washed with phosphate-buffered saline (PBS, pH 7.4) to remove the non-adherent cells. Different concentrations of the test compounds in mLB were added to the wells of 96-well microtiter plates and incubated for another 24 h at 30 °C. Then, crystal violet staining was performed, as described above, and absorbances were measured at OD_570_ [35]. The results are presented as the means of at least three independent cultures.

### 2.5. Confocal Laser Scanning Microscopy (CLSM) and COMSTAT Analysis

The inoculum of *V. parahaemolyticus* was grown in 96-well polystyrene plates (SPL life sciences, Pocheon-si, South Korea) without shaking at 30 °C for 24 h in the presence or absence of CNMA and its selected derivatives (50 µg/mL). The formed biofilms were stained with 100 μL of pre-warmed PBS containing CFSE (carboxyfluorescein diacetate succinimidyl ester) (Invitrogen, Eugene, OR, USA) for 20 min at 37 °C and were visualized by CLSM (Nikon Eclipse Ti, Nikon Instruments, Tokyo, Japan), as described by [37]. At least 10 random positions in each of the three independent cultures were chosen for microscopic analysis [38]. Biofilm formation was quantified using COMSTAT biofilm software [39] to determine the biomasses (µm^3^/µm^2^), mean thicknesses (µm), and substratum coverage (%).

### 2.6. Scanning Electron Microscopy (SEM)

The effects of CNMA and its derivatives on the morphological characteristics of *V. parahaemolyticus* cells and biofilm were examined by SEM (S-4200, Hitachi, Tokyo, Japan), as previously described [37,40]. Briefly, a nylon membrane was cut into 0.5 × 0.5 cm pieces, placed in 96-well plates containing *V. parahaemolyticus* grown at static condition with or without test compounds at 50 µg/mL and incubated for 24 h at 30 °C. The cells adhered to membranes were fixed with 2.5% glutaraldehyde and 2% formaldehyde overnight, and post-fixed using PBS and osmium tetroxide. This was followed by dehydration using a graded series of ethanol (30, 50, 70, 80, 90, 95, and 100%; 15 min each) and isoamyl acetate. Subsequently, a critical-point dryer (Hitachi HCP-2, Tokyo, Japan) was used for dehydration without damaging the structure of the cells on the filter, sputter-coated with palladium/gold, and examined using SEM at magnifications ranging from ×5000 to 20,000 at an accelerating voltage of 15 kV.

### 2.7. Analysis of Swimming and Swarming Motility

For the swimming assay, 1 µL of an overnight culture of *V. parahaemolyticus* cell was inoculated on the center of the semi-solid mLB plate (containing 0.3% Neogen LB agar) in the presence or absence of CNMA and its derivatives. For the swarming assay, 1 μL of the overnight culture was dropped on the solid mLB plate (containing 0.6% agarose). The semi-diameter of the area covered by the bacterial cells was measured and images were taken after incubation at 30 °C for 24 h. The freshly prepared media used were dried for 4 h and incubated at the upright position after inoculation [41,42]. The experiments were carried out with three independent cultures and six replicates.

### 2.8. Yeast Agglutination Assay

The effects of CNMA and its derivatives on the *V. parahaemolyticus* fimbria activity were investigated using *Saccharomyces cerevisiae* (Sigma-Aldrich, St. Louis, MO, USA), as reported previously [43]. Briefly, approximately 0.4 mL of the adjusted *V. parahaemolyticus* cells (OD 0.5) was added to 14 mL tubes containing 1.5 mL of PBS and 0.5 mL *S. cerevisiae* (2% *w*/*v* in PBS). The initial OD_600_ of the mixture was achieved by gentle vortexing for 5 s and measured using a spectrophotometer. After incubation for 10 min at room temperature, 100 µL of the upper phase was transferred to a 96-well plate, and the final OD_600_ was measured. The presence of visible aggregates of agglutinated cells affected the OD_600_ measurement. Therefore, vigorous vortexing for 30 s disturbed the agglutinated cells before taking the final OD_600_ values. The agglutination was expressed in percentage using the equation: $100\times \left(\frac{1-{\mathrm{OD}}_{600}\;\mathrm{before}\;\mathrm{vortexing}}{{\mathrm{OD}}_{600\;}\mathrm{after}\;\mathrm{vortexing}}\right)$.

### 2.9. Bacterial Surface Hydrophobicity Assay

*V. parahaemolyticus* surface hydrophobicity was determined by microbial adhesion to hydrocarbon [44,45]. Briefly, the test bacterium (1:100) was grown in mLB broth at 30 °C for 24 h at 250 rpm with or without CNMA and its derivatives. After incubation, the tubes were centrifuged at 11,000× *g* for 15 min at 4 °C. The supernatant was discarded, and the pellets were washed twice with sterile PBS (pH 7.2) and resuspended in the same buffer to adjust cells to an optical density (OD_600_) of approximately 0.5 (A_o_). Subsequently, 4 mL of the adjusted cell was dispensed into glass tubes containing 1 mL of xylene. The mixture was vortexed vigorously (1 min) and left to settle for 30 min until the aqueous phase was separated from the organic phase. The absorbance of the aqueous phase was measured at 600 nm (A_i_) to determine the cells remaining. The percentage of hydrophobicity was calculated using the following equation: $\mathrm{Hydrophobicity}\;\left(H\right)\%=\frac{\left({\;A}_{o}-{\;A}_{i}\right)}{A_{o}}\times 100$. Xylene was used because of its higher hydrophobicity than the other hydrocarbons. The results presented are the means of measurements taken from three independent cultures with six replicates.

### 2.10. Exoprotease Assay

The effects of CNMA and its derivatives on extracellular protease production by *V. parahaemolyticus* were determined as reported previously [43]. Briefly, the test bacterium was diluted (1:100) in mLB and treated with varying concentrations of the test compounds for 24 h at 30 °C and 250 rpm. The overnight treated culture was centrifuged at 15,000× *g* for 15 min. Subsequently, 125 µL of 2% (*w*/*v*) azocasein was added to 75 µL of the supernatant and kept at 37 °C for 30 min. A 10% trichloroacetic acid solution (600 µL) was introduced to stop the proteolysis. The unreacted azocasein was precipitated at −20 °C for 30 min, and the precipitates were separated by centrifugation at 10,000× *g* for 10 min. Approximately 600 µL of the supernatant was aliquoted and mixed with NaOH (700 µL), and the absorbance was read at 440 nm. The experiment was carried out with three independent cultures.

### 2.11. Indole Production Assay

The effects of pH, CNMA, and its derivatives on indole production were investigated as described previously with a slight modification [46,47,48]. Briefly, the cells of *V. parahaemolyticus* were grown in the presence or absence of test compounds and incubated at 30 °C with shaking at 250 rpm for 10 h. Then, 1 mL of grown culture was taken and centrifuged at 11,300× *g* for 5 min and the supernatant was mixed with 300 µL of Kovacs reagent (10 g of *p*-dimethyl amino benzaldehyde dissolved in 50 mL of HCl and 150 mL of amyl alcohol). The mixture was left at room temperature for 2 min. After this, 50 µL of the mixture was aliquoted and added to 1 mL of a HCl-amyl alcohol solution (mixture of 75 mL of HCl and 225 mL of amyl alcohol). For the effect of pH on indole production, the medium at pH 7 initially was adjusted to pH 5 and pH 9 with 35% HCl and 5 N NaOH, respectively, before inoculation. The extracellular indole was measured at 540 nm, and the concentration was determined using a standard curve prepared from indole in the range of 0.1–0.8 mM.

### 2.12. Biotic Surface Assay

The efficacy of CNMA derivatives on the seafood surface was investigated, as described previously, with some modifications [49]. Briefly, the main body of the squid, called the mantle, was gently separated from the peripheral parts, such as the tentacles and hood. The mantles were placed in sterile Petri dishes and sliced into equal sizes (1.5 cm × 1.5 cm × 0.2 cm) using a sterile scalpel. The samples were washed several times with distilled water, dried in a safety cabinet (JSCB-1200SB, JSR, Gongju, Korea) for approximately 1 h, and grouped into different categories. The blank control included the squid surface without inoculation and treatment, while the untreated control was inoculated with 1 mL (1 × 10^6^ CFU/mL) of *V. parahaemolyticus* culture. The other groups were treated with 50 µg/mL of CNMA and its analogs. The samples were incubated at 30 °C for 24 h without shaking to allow growth and biofilm formation on the squid surfaces. After incubation, the samples were prepared for SEM analysis as described above. None of the sample surfaces were exposed to disinfecting agents to determine the effect of treatment on the background microflora.

### 2.13. RNA Isolation and Quantitative Reverse Transcriptase PCR (qRT-PCR)

For transcriptomic studies, an overnight culture of *V. parahaemolyticus* was reinoculated into 25 mL of LB broth at 30 °C in 250 mL flasks to achieve the OD_600_ of 1.0 and incubated for 4 h more with shaking at 250 rpm in the presence or absence of 4-BromoCNMA, 4-ChloroCNMA, and 4-NitroCNMA (50 µg/mL). RNase inhibitor (RNAlater, Ambion, TX, USA) was added, and the cells were chilled immediately for 30 s in a dry ice bath with 95% ethanol to prevent RNA degradation. The cells were harvested by centrifugation at 16,600× *g* for 5 min at 4 °C, and the total RNA was isolated using a Qiagen RNeasy mini-Kit (Valencia, CA, USA). qRT-PCR was used to examine the expression of various virulence genes related to QS and biofilm formation (*aphA*, *cspA*, *luxI*, *luxS*, *mshA*, *opaR*, *oxyR*, *tnaA* and *qsvR*), motility (*fliA*), virulence (*tdh* and *vopS*), multidrug efflux (*vmrA* and *vmeB*), and membrane integrity (*ef-Tu*, *fadL* and *nusA*). The analysis was carried out as previously described [50] using SYBR Green master mix (Applied Biosystems, Foster City, CA, USA) and an ABI StepOne Real-Time PCR System (Applied Biosystems). At least two independent cultures with three repetitions were used. The test compound did not affect the expression of the housekeeping gene (*16S rRNA*). Table S2 presents the primer sequence of the genes used for this study.

### 2.14. Statistical Analysis

The experiments were carried out with three independent cultures and six repetitions, at least in triplicate. The data presented are the average values ± SD. The differences between the means were tested using a Student *t*-test. The differences were considered significant at *p* ≤ 0.05.

## 3. Results

### 3.1. Biofilm Inhibitory and Antimicrobial Activities of Cinnamaldehyde (CNMA) and Its Derivatives against V. parahaemolyticus and V. harveyi

In this study, cinnamaldehyde and ten of its derivatives were screened for their antibacterial and antibiofilm potential against *V. parahaemolyticus* 17802 and *V. harveyi* 14126 at concentrations of 20 and 100 µg/mL. Of the compounds tested, 4-BromoCNMA, 4-ChloroCNMA, 4-NitroCNMA, and 2-MethoxyCNMA had significant inhibitory effects on the *V. parahaemolyticus* biofilms at 100 µg/mL compared to a non-treated control with percentage biofilm inhibition of 99.6, 98.9, 98.7, and 61.8%, respectively (Figure 1B). The backbone CNMA at the same concentration showed a weak effect on biofilm formation. Furthermore, CNMA also had a slight inhibitory effect on biofilm formation in *V. harveyi*, while its derivatives—CNMA oxime, 4-BromoCNMA, 4-ChloroCNMA, 4-FluoroCNMA, and 4-NitroCNMA—exhibited marked effects with percent reduction in biofilm formation of 57.0, 100.0, 100.0, 75.0, and 99.7 at 100 µg/mL, respectively (Figure 1C). This result showed that CNMA derivatives were active against the biofilm formation of *V. parahaemolyticus* and *V. harveyi* (Figure 1B,C).

The MICs of CNMA and its derivatives against the planktonic cells of *V. parahaemolyticus* and *V. harveyi* were found to be in the range 50 to >500 µg/mL under static conditions (Table S1). In particular, the MICs of 4-BromoCNMA, 4-ChloroCNMA and 4-NitroCNMA were the lowest concentration at 50 µg/mL, while 4-DimethylaminoCNMA, CNMA oxime, and α-MethylCNMA with the highest MICs could not inhibit *V. parahaemolyticus*, even at 500 µg/mL. The backbone CNMA had moderate antimicrobial activity at 200 µg/mL. Furthermore, growth curve analysis was carried out and confirmed that CNMA analogs significantly prevented the growth of the test bacterium at the concentration of 50 and 100 µg/mL. Compared to the untreated control, there was not much alteration in the growth curve of *V. parahaemolyticus* treated with backbone CNMA at concentrations of 20–100 µg/mL, (Figure 1D). Similarly, the antibacterial activities of CNMA and its derivatives against another species, *V. harveyi* ATCC 14126, showed a similar trend to that of *V. parahaemolyticus* (Table S1). Hence, the CNMA derivatives appear to be potential broad-spectrum antimicrobial agents against members of *Vibrionaceae*.

A more detailed biofilm study showed that CNMA and three CNMA analogs dose-dependently inhibited *V. parahaemolyticus* biofilm formation (Figure S1A). For example, CNMA at 200 µg/mL significantly inhibited biofilm formation while 4-BromoCNMA, 4-ChloroCNMA, and 4-NitroCNMA at 50 µg/mL inhibited it by more than 95% (Figure S1A). Since the two major approaches to treat biofilms are to prevent their formation or eradicate already established biofilms [51], the ability of the compounds to disrupt preformed biofilms was investigated. CNMA and its derivatives at 200 or 400 µg/mL (four to eight times higher concentrations than biofilm inhibition) could disrupt established biofilms (Figure S1B). This result shows that biofilm dispersal is more difficult than biofilm inhibition.

Among the four hits with higher antibiofilm activities against *V. parahaemolyticus*, 4-ChloroCNMA, 4-NitroCNMA, and 4-BromoCNMA were assessed further because of their observed lower minimum inhibitory concentrations against the planktonic cells, while the backbone CNMA was used as a structural control.

### 3.2. Light, Confocal and Electron Microscopy Observation of Biofilm Inhibition by CNMA Derivatives

**Figure 2:**
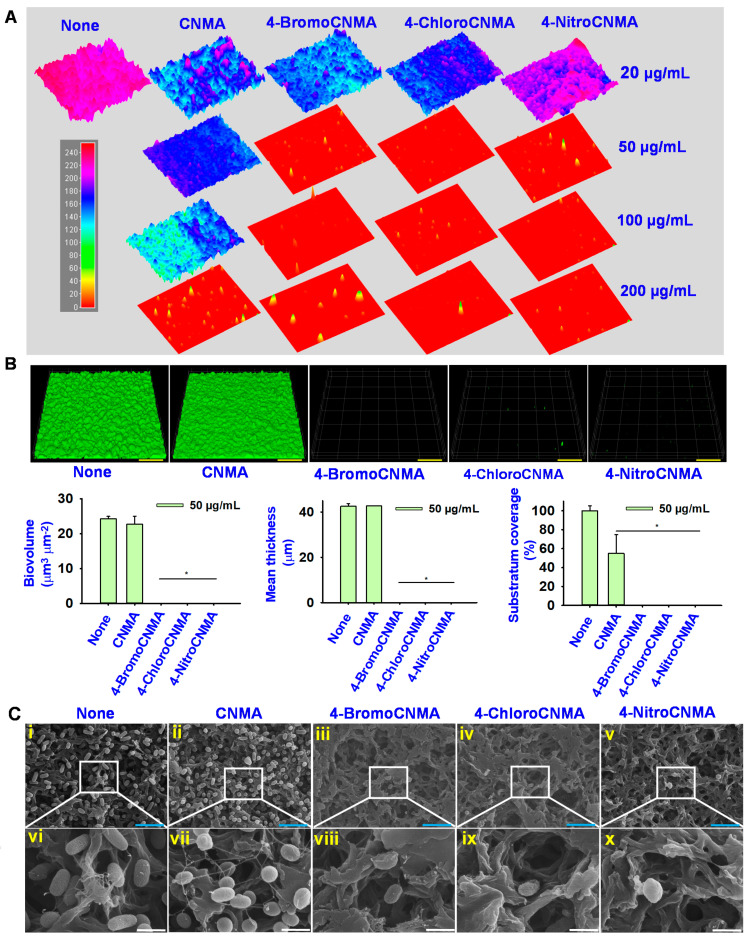
Antibiofilm activity of three selected CNMA derivatives. Three-dimensional optical microscopy images of biofilm formation with/without treatment (**A**), CLSM and COMSTAT analysis of biofilm images with or without 50 µg/mL of CNMA, 4-BromoCNMA, 4-ChloroCNMA, and 4-NitroCNMA (**B**). The yellow bar represents 100 µm. SEM images showing the effects of CNMA derivatives on cell and biofilm morphology (**C**). The blue and white scale bars are 6 and 1.5 µm, respectively. * denotes a significant difference at *p* < 0.05.

Antibiofilm efficacies of CNMA derivatives were further affirmed using microscopic methods (Figure 2). The optical and confocal images revealed strong biofilms, which are distributed uniformly, densely structured with complete surface coverage, in the control (Figure 2A,B). In contrast, the group treated with 50 µg/mL of 4-BromoCNMA, 4-ChloroCNMA, and 4-NitroCNMA completely inhibited biofilm development. The confocal images were subjected to COMSTAT analysis (Figure 2B) to quantify the impact of the test compounds, and it showed that the three derivatives completely halted the aggregation of biofilm cells as no biomass, thickness, and surface coverage were visible. For example, in untreated controls, *V. parahaemolyticus* formed dense biofilms (biovolume > 25 µm^3^µm^−2^, thickness > 40 µm and 100% surface coverage), whereas the three CNMA analogs at 50 µg/mL reduced the biofilm density, thickness, and surface coverage substantially by more than 98%.

In addition, SEM analysis revealed that the untreated culture retains a dense biofilm with typical normal rod shape of *V. parahaemolyticus* entrapped within the matrix (Figure 2C). On the other hand, there was a visible reduction in cell aggregation and the number of microcolonies observed after treatment with 50 µg/mL of 4-BromoCNMA, 4-ChloroCNMA, and 4-NitroCNMA, indicating their antibiofilm activities. Similarly, the antibacterial activity was evident as the majority of the biofilm cells lost their typical rod shape to form a wrinkled round shape after a treatment with CNMA derivatives, which is suggestive of cytoplasmic content leakage. The bacterial cell membrane is a critical barrier that ensures the sustenance of cellular energy by maintaining ion homeostasis, and a slight change or damage can adversely affect the metabolism, leading to the death of bacteria [52]. This is the first study to report the effects of CNMA derivatives on the biofilm and cell morphology of *V. parahaemolyticus*.

### 3.3. CNMA Derivatives Reduced Surface Motility, Fimbriae, Hydrophobicity, and Protease Production

**Figure 3:**
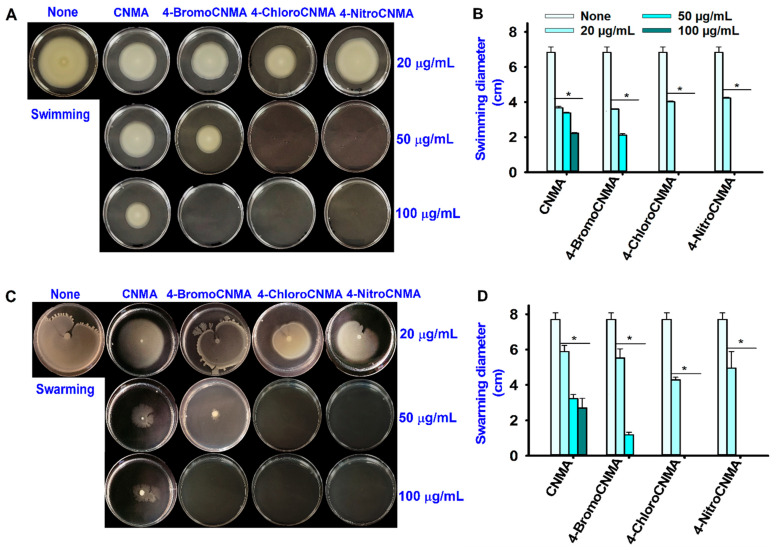
Effects of CNMA derivatives on the surface motility of *V. parahaemolyticus*. Swimming motility (**A**), swarming motility (**C**), diameter of migration in the swimming assay (**B**), diameter of migration in the swarming assay (**D**). * denotes a significant difference at *p* < 0.05.

Bacterial virulence factors remain attractive targets for drug development [53]. This study examined the effects of CNMA and its derivatives on various virulence factors of *V. parahaemolyticus* to understand the possible antibiofilm mechanism of action. Swimming and swarming motilities are considered critical virulence factors enhancing the colonization of host surfaces or target organs by pathogenic *Vibrios* [54,55]. Therefore, the ability of CNMA and its selected derivatives to inhibit motility was studied (Figure 3). Compared to the control, CNMA decreased the swimming and swarming abilities of *V. parahaemolyticus* dose-dependently, while 4-BromoCNMA restricted them noticeably at 50 µg/mL and with total inhibition at 100 µg/mL. On the other hand, 4-ChloroCNMA and 4-NitroCNMA restricted the swimming and swarming phenotypes at 50 and 100 µg/mL (Figure 3A,C). Quantitatively, the bacterial motility diameters decreased with increasing doses (Figure 3B,D).

**Figure 4:**
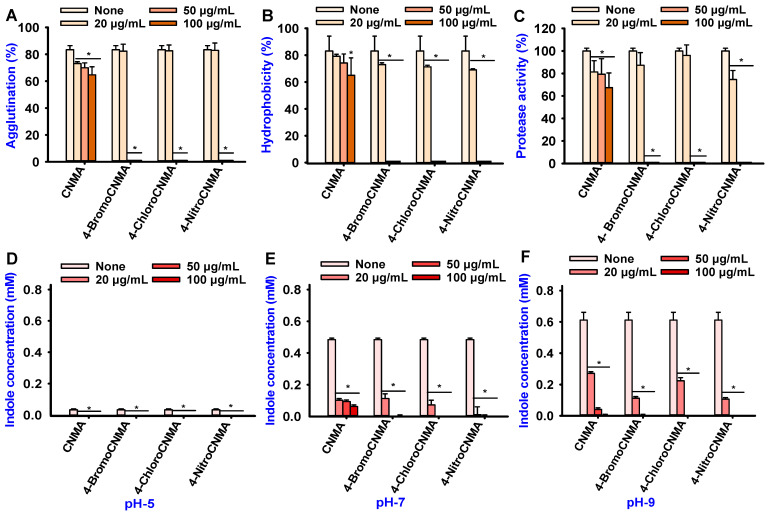
Effect of CNMAs on the virulence factors. Fimbriae mediated yeast agglutination (**A**), hydrophobicity (**B**), and protease activity (**C**) of *V. parahaemolyticus*. Indole production in *V. parahaemolyticus* at pH 5 (**D**), pH 7 (**E**), and pH 9 (**F**). The error bars and asterisks (*) represent standard deviations and significant differences (*p* < 0.05), respectively, vs. the non-treated controls.

Fimbriae on bacterial cell surfaces play key roles in biofilm formation on both abiotic and biotic surfaces [56]. In particular, type 4 fimbriae played a key role in the attachment and persistence of *Vibrio* spp. in oysters [56]. Therefore, this study investigated the effect of CNMA and its derivatives on fimbriae using yeast agglutination (Figure 4A). CNMA was observed to suppress fimbriae production in a dose-dependent manner. Compared to the control, no significant changes were observed with its derivatives (4-BromoCNMA, 4-ChloroCNMA, and 4-NitroCNMA) at 20 µg/mL, while at higher concentrations of 50 and 100 µg/mL, Fimbriae were inhibited drastically by the derivatives.

The cell surface hydrophobicity (CSH) is a key feature for adhesion and biofilm formation [57]; regulating it is worth the effort. In this study, CNMA had concentration-dependent inhibition on the hydrophobic cells of *V. parahaemolyticus*, whereas its derivatives exhibited a moderate effect at 20 µg/mL and a complete reduction at 50 and 100 µg/mL (Figure 4B).

*V. parahaemolyticus* used protease to exert cytotoxic effects on CHO and vero cells [58]. Therefore, this study examined the impact of CNMA and its analogs on protease production. CNMA produced a slight but significant dose-dependent suppression of protease, while its derivatives showed complete inhibition at 50 and 100 µg/mL (Figure 4C). Although CNMA had a weak effect on biofilm inhibition in *V. parahaemolyticus* at lower concentrations tested (Figure 1B), it dose-dependently inhibited the fimbriae, hydrophobicity, and protease activity, which are associated with the development of biofilms and infections.

### 3.4. CNMA and Its Derivatives Suppressed Indole Production by V. parahaemolyticus

*V. parahaemolyticus* is an established indole producer [5]. Recently, indole was recognized as a new quorum-sensing signal molecule that regulates various bacterial phenotypes, including resistance to antibiotics and biofilm formation [59]. The pH of the medium affects the expression of *tnaA* responsible for indole production in bacteria [48,60,61]. Therefore, the effects of CNMA and its derivatives were examined on indole production under different pHs. As revealed by the untreated control, *V. parahaemolyticus* produced 0.05 mM, 0.5 mM and 0.6 mM indole at pH 5, pH 7 and pH 9, respectively (Figure 4D–F). This suggests that pH plays a vital role in indole production by *V. parahaemolyticus*. In particular, at pH 5, CNMA and its three derivatives inhibited indole secretion (Figure 4D). The effects of CNMA and CNMA derivatives were more profound at neutral and high pHs. Specifically, 4-BromoCNMA, 4-ChloroCNMA, and 4-NitroCNMA at 50 µg/mL completely inhibited indole production (Figure 4E,F).

### 3.5. CNMA Derivatives Eradicated V. parahaemolyticus on Squid Surface

**Figure 5:**
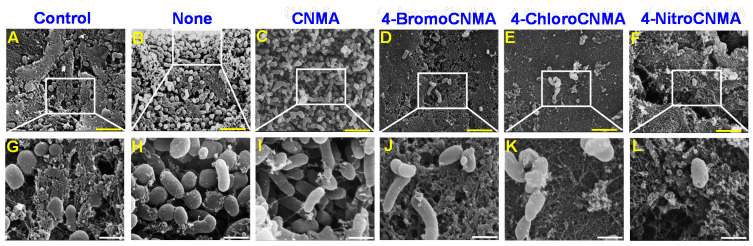
SEM images of squid surface infected with *V. parahaemolyticus* and treated with or without CNMA and its analogs. The control represents squid surface without *V. parahaemolyticus* (**A**,**G**) while none is squid surface + *V. parahaemolyticus* without treatment (**B**,**H**) and the treated groups; CNMA (**C**,**I**), 4-BromoCNMA (**D**,**J**), 4-ChloroCNMA (**E**,**K**), 4-NitroCNMA (**F**,**L**). Yellow and white scale bars are 6 µm and 1.5 µm, respectively.

*V. parahaemolyticus* and other aquatic bacteria are colonizers of seafood surfaces. They persist on the surfaces of marine organisms or food processing environments, leading to the formation of bacterial biofilms [49]. Therefore, this study examined the ability of CNMA derivatives to prevent growth and biofilm formation by *V. parahaemolyticus* and the background microflora using SEM imaging (Figure 5). In the blank control, only background microflora was observed (Figure 5A,G) while the untreated (None) and CNMA treated groups showed mixed microorganisms of *V. parahaemolyticus* and the existing microbial flora (Figure 5B,C,H,I). On the other hand, 50 µg/mL of 4-BromoCNMA, 4-ChloroCNMA, and 4-NitroCNMA eliminated biofilms of the test bacterium and drastically reduced the background flora (Figure 5D–F,J–L). This supports the potential of CNMA analogs as broad-spectrum antibacterial agents for controlling microbes on seafood surfaces.

### 3.6. CNMA Derivatives Repressed the Expressions of Biofilm, Quorum Sensing, and Other Virulence-Related Genes

**Figure 6:**
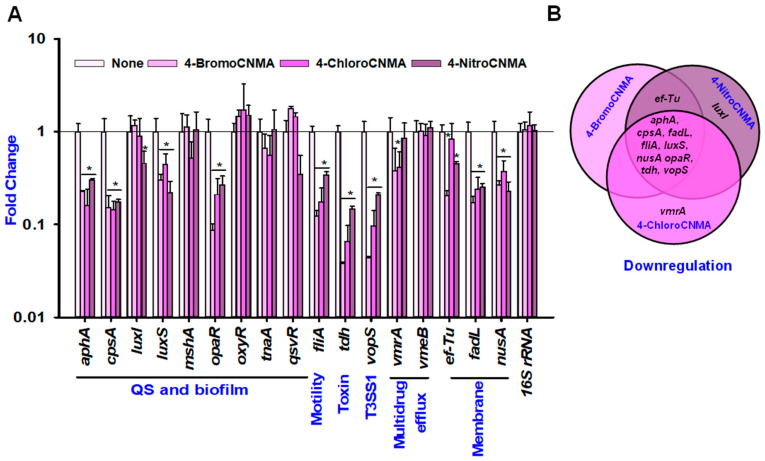
Transcriptional profiles of *V. parahaemolyticus* treated with or without 4-BromoCNMA, 4-ChloroCNMA, and 4-NitroCNMA. Relative expressions represent transcriptional levels after treatment with the CNMA derivatives at 50 µg/mL compared to non-treated controls (**A**). The bars represent the mean ± SDs of two independent cultures performed in triplicate. The *16S rRNA* gene was used as a housekeeping gene. The Venn diagram represents the downregulation of genes by the CNMA derivatives (**B**). * denotes a significant difference at *p* < 0.05.

The possible molecular mechanisms responsible for the antibacterial and antivirulence effects of the three CNMA analogs were examined by transcriptomic analysis to determine the changes in the expression of 17 virulence- and biofilm-related genes in *V. parahaemolyticus*. As observed in Figure 6, three CNMA analogs commonly altered the expression of nine essential genes, such as *aphA*, *cpsA*, *fadL*, *fliA*, *luxS*, *nusA*, *opaR*, *tdh*, and *vopS*. For example, three CNMA analogs at 50 µg/mL significantly downregulated the major QS and biofilm-related genes (*aphA*, *cpsA*, *luxS*, and o*paR*) in *V. parahaemolyticus* by different folds. In particular, 4-BromoCNMA, 4-ChloroCNMA and 4-NitroCNMA repressed the genes by (4-, 7-, 3-, 12-), (6-, 7- 3-, 5-) and (3-, 6-, 5-, 4-) fold, respectively. Similarly, the other genes related to motility (*fliA*), thermostable hemolysin (*tdh*), secretion system (*vopS*) and membrane (*fadL* and *nusA*) were also downregulated by 4-BromoCNMA (8-, 26-, 22-, 6- and 4-fold), 4-ChloroCNMA (6-, 15-, 10- 4-, and 4-fold) and 4-NitroCNMA (3-, 7-, 5-, 4- and 3-fold). On the other hand, these analogs differentially affected the expression of the other genes. While 4-BromoCNMA and 4-NitroCNMA downregulated the elongation factor (*ef*-*Tu*) by 5- and 2-fold, only 4-NitroCNMA could repress *luxI*. However, the expression of type IV pilin gene (*mshA*) related to biofilm formation, the tryptophanase gene (*tnaA*) related to indole production, QS and biofilm (*qsvR* and *oxyR*) and multidrug efflux genes (*vmeB* and *vmrA*) were not affected significantly by the three CNMA analogs (Figure 6).

## 4. Discussion

*Vibrio* spp. in biofilm mode develop adaptation to various stress conditions and antibiotics, and unavoidably increase multidrug resistance strains [31]. Inhibiting this biofilm lifestyle and other virulence factors could attenuate bacterial pathogenicity, and enable the host immune system to successfully clear the pathogen [62].

In this study, 4-BromoCNMA, 4-ChloroCNMA, 2-MethoxyCNMA, and 4-NitroCNMA exhibited high inhibitory activities against *V. parahaemolyticus* biofilm (Figure 1B). This is consistent with earlier studies on *V. anguillarum* LMG 4411 [3] and *S. pyogenes* biofilms with an evident decrease in its biomass and average thickness [28]. On the other hand, 2-NitroCNMA and 4-MethoxyCNMA did not affect *V. vulnificus* LMG 16867 biofilm formation [3]. Similar to α-BromoCNMA and cinnamic acid-mediated eradication of *E. coli* MG 1655 persister cells [23] and preformed biofilms of *S. aureus* and *E. coli* [63], the CNMA and its derivatives also dispersed preformed biofilms of *V. parahaemolyticus* (Figure S1B). Consequently, this suggests that tested CNMA analogs could penetrate the extracellular polymeric substance matrix, disrupt it and distort the embedded cells (Figure 2C) as observed by SEM, since the EPS matrix generally hinders the penetration of drug [42].

The antibacterial activity displayed by CNMA (Figure 1D and Table S1) is consistent with a report that it significantly inhibited the growth of *E. coli* ATCC 33456, *P. aeruginosa* PAO1, *P. putida* KT2440, and *P. fluorescens* pSMC21 [64]. In addition, the potential of CNMA derivatives to inhibit the cell growth of *V. parahaemolyticus* at lower MICs corroborates the previous study [3]. The lower MICs (Table S1) show that a smaller amount can combat the test bacteria and reduce the risks of drug resistance development. Additionally, it suggests that the antibacterial properties contributed to the antibiofilm efficacy displayed by these compounds. Previous reports showed that combined use of agents with both antibiofilm and antimicrobial properties enhanced bactericidal activity and often reduced biofilm formation more effectively than when administered individually [65,66]. Therefore, CNMA derivatives with both properties in this study may serve as a great alternative in combating biofilm-mediated infections.

Notably, the three analogs showed a similar antibiofilm and antimicrobial trend against *V. parahaemolyticus* and *V. harveyi* (Table S1 and Figure 1B,C). It appears that CNMA derivatives are potential broad-spectrum antibiofilm agents against members of *Vibrionaceae*. This is partially supported by a previous report that CNMA derivatives inhibited biofilm formation in *V. vulnificus* LMG 16867 and *V. anguillarum* LMG 4411 [3]. The ability of the analogs to eradicate the cells of the *V. parahaemolyticus* and reduction in the background flora on squid surface in this study (Figure 5) further confirm the preservative efficacy of CNMAs in inactivating foodborne pathogens and nonpathogenic spoilage organisms that affect the shelf life of seafoods [67]. On the other hand, the inhibitory effect on the background microbes might also affect beneficial flora. Due to this reason and the hydrophobic nature of CNMAs, future application for infection control in aquaculture may be impaired. Therefore, nano or film-based encapsulation or emulsion can be explored to ensure controlled delivery of the active CNMAs within the animal system as well as minimizing toxic effects [68,69].

The inhibition of motility by CNMA used as structural control in this study confirmed earlier studies that cinnamaldehyde inhibited both the motility phenotypes in *V. parahaemolyticus* [31], and *E. coli* ATCC 33456 [64]. 4-BromoCNMA, 4-ChloroCNMA, and 4-NitroCNMA exhibited greater inhibitory activities than the backbone compound, CNMA (Figure 3). This result suggests that these derivatives might have exerted their antibiofilm potentials partially by disrupting the *Vibrio* motility and subsequently preventing their ability to reach the surfaces. Because the motility phenotypes are regulated by the QS pathways [31], their inhibitions by CNMA derivatives further corroborate an earlier report that CNMA and its derivatives reduced virulence by interfering with the DNA binding ability of the QS response regulator, LuxR [3].

Fimbriae mediate an array of other virulence functions, including gene transfer, motility, bacterial aggregation, and adherence essential in bacterial pathogenesis [53]. The suppression of fimbriae production by CNMA and its derivatives in this study (Figure 4A) corroborates a previous finding that it reduced Fimbriae production in enterohemorrhagic *E. coli* O157:H7 [25]. The observed inhibition implies the potential of CNMA derivatives to interfere with the virulence activities attributed to fimbriae.

Previously, a chromone derivative, CM3a, eradicated the *S. aureus* biofilms by inhibiting cell adherence [70]. In this study, CNMA and its derivatives—4-BromoCNMA and 4-ChloroCNMA—reduced CSH at sub-inhibitory concentrations of 100, and 20 µg/mL, respectively. This is contrary to a report that it increased the surface hydrophobicity in *Streptococcus mutans* [45]. Although the reason for this difference is unknown, it might be bacterial species dependent. Furthermore, Fimbriae were reported to enhance CSH because they contained a high amount of hydrophobic amino acid residues [71]. Therefore, the inhibition of these attributes highlights the potential of the analogs to minimize the adhesion of *V. parahaemolyticus* to host and seafood surfaces. This provides a better alternative and increases the shelf life of packaged seafood products rather than eradicating already formed biofilms.

Furthermore, exoproteases, which play important roles in the virulence of clinical *V. parahaemolyticus* isolate [72], were strongly inhibited by CNMA derivatives in this study (Figure 4C). This is consistent with the finding of [3] where 2-NitroCNMA and 4-MethoxyCNMA decreased the protease activity in *V. anguillarum* LMG 4411. In the same study, CNMA was more active against *V. anguillarum* than the majority of its derivatives [3]. However, CNMA derivatives had more inhibitory potentials against protease secretion in *V. parahaemolyticus* in this study. Previously, the proteases Vvp in *V. vulnificus* and EmpA in *V. anguillarum* were involved in colonizing the mucosal surfaces and virulence during infection of salmon and hemorrhagic skin damage, respectively [73,74,75]. Therefore, the inhibition of protease could have a deleterious effect on the ability of *V. parahaemolyticus* to initiate an infection.

Indole production was reported in different bacteria that carry a copy of the tryptophanase (*tnaA*) gene [46]. While this is the first study to report the effect of pH on indole production in *V. parahaemolyticus*, we established that indole synthesis in the test bacterium is affected by pH. The observation that *V. parahaemolyticus* negligibly produces indole at low pH (Figure 4D) and high amounts at high pH (Figure 4F) agrees with previous findings that an acidic pH inhibited indole production in *E. coli*, while an alkaline pH increased it [48]. This was attributed to the repression [76] and induction [61,77] of *tnaA* gene expression under acidic and alkaline conditions, respectively. Interestingly, the amount of extracellular indole produced by *V. parahaemolyticus* (0.6 mM) at high pH in this study (Figure 4F) was previously reported for both *E. coli* and *V. cholerae* [47,78]. This indicates that *V. parahaemolyticus* is another major producer of quorum-sensing molecule indole. Therefore, its inhibition by CNMA and its derivatives suggests that they could interrupt the indole-signaling pathway in *V. parahaemolyticus* and intercepting these pathways is an attractive antivirulence therapeutic strategy.

Previously, CNMA derivatives, such as α-bromoCNMA, displayed a mechanism of action independent of reactive oxygen species against *E. coli* [23]. They also reduced virulence by interfering with the DNA binding ability of the QS response regulator *LuxR* as reported in *V. anguillarum* [3]. In this study, the treatment of *V. parahaemolyticus* with the three analogs of CNMA downregulated nine genes related to biofilm formation and QS (*aphA*, *cpsA*, *luxS*, and *opaR*), virulence (*fliA*, *tdh*, and *vopS*) and membrane (*fadL* and *nusA*) (Figure 6).

The major QS regulators, AphA and OpaR of *V. parahaemolyticus*, act as the mediators of virulence and biofilm formation during host colonization and infection [79,80]. Similarly, *cpsA* is crucial for the production and transportation of capsular polysaccharides (a component of *V. parahaemolyticus* EPS) [81]. Therefore, the downregulation of these genes could result in a loss of surface adherence, biofilm-forming, and virulence abilities that can be linked with the reduced phenotypic characteristics. Additionally, the repression of *fliA*—a flagella assembly gene—explains the inhibition of CSH (Figure 4B), swimming and/or swarming (Figure 3A,C) motilities. In addition, *vopS* secreted by type III secretory system 1 during infection and used for delivering effectors into host cells was significantly repressed. On the other hand, CNMA derivatives inhibited extracellular indole production but could not considerably repress *tnaA*. Since indole is a cell–cell communication signal [82], we opined that it might be a part of the quorum-sensing network in *V. parahaemolyticus*, and the downregulation of other major QS genes may have interfered with the phenotypic indole production. Furthermore, the *ef-Tu* gene encodes an elongation factor involved in extending the peptide chain during protein synthesis. Hence, its downregulation could halt protein synthesis and cell elongation, resulting in a distortion of the rod shape of the cells, as observed by SEM (Figure 2C). Similarly, *nusA*, which is critical to the viability of *V. parahaemolyticus* cells and DNA damage repair [83], was downregulated. This may interfere with the ability of the cell to perform these functions and, subsequently, cause cell death. The *fadL* gene, a fatty acid transport protein, is significantly related to membrane integrity and fatty acid production. Its repression might have altered the membrane and the cell structure.

**Figure 7:**
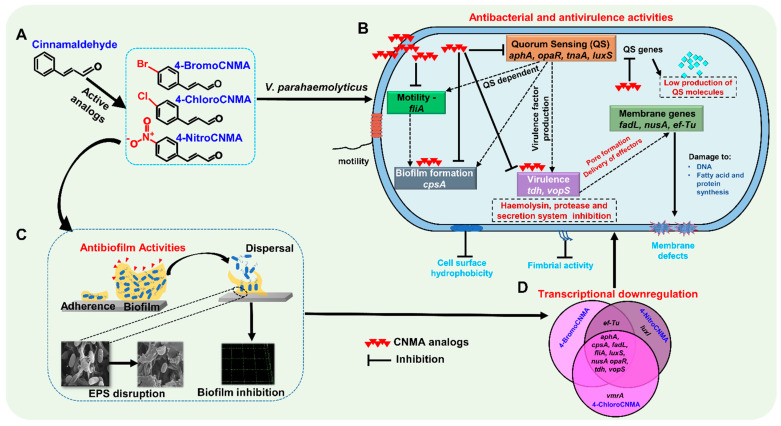
Proposed mechanism of antibacterial, antibiofilm and antivirulence actions of CNMA derivatives against *V. parahaemolyticus*. Active CNMA analogs (**A**) violate the cell wall as well as interfere with quorum-sensing regulators, biofilm formation, surface adherence factors, motility, protease, toxin production, type III secretion system, and DNA repair in *V. parahaemolyticus* (**B**,**C**). It is envisaged that the repression of responsible QS and virulence genes (**D**) explains the mechanism behind the antimicrobial efficacies of CNMA analogs.

Overall, as observed in this study, CNMA and its derivatives in a previous finding inhibited biofilm formation and reduced stress survival ability as well as pigment/protease production in some *Vibrio* spp. This was attributed to the ability of these compounds to decrease the DNA binding ability of the LuxR [3]. Similarly, furanones were originally thought to competitively inhibit the binding of autoinducers (AIs) to their receptors, but it is now established that they destabilize the LuxR in *V. fischeri* and *V. harveyi*. This affects the ability of LuxR to bind DNA and initiate transcription [84]. The OpaR remains the major QS regulator in *V. parahaemolyticus*, and it is homologous to LuxR QS regulator in *V. harveyi* [85,86]. Previously, CNMA was reported to effectively inhibit both autoinducer-1- (AHL) and autoinducer-2-mediated QS [87]. Based on these previous studies, it appears that CNMA derivatives manipulate the QS systems to exert antipathogenic properties. However, we are not clear on the mechanism underlying the activities of these derivatives against *V. parahaemolyticus*. Therefore, we speculate that CNMA derivatives employ a similar non-competitive mode of QS signal disruption to carry out their antimicrobial activities. In addition, since CNMAs suppressed *Vibrio* response to stress condition [3], cell survival inhibition may also be partially responsible for the activity (Figure 7).

In this study, the various antimicrobial efficacies exhibited by CNMA derivatives might be attributed to their structural characteristics. Previously, the structural activity relationship of coumarin-based derivatives showed that substitution with electron-withdrawing groups (–Cl, –NO_2_, and –Br) enhanced the antibacterial activity [88]. Similarly, halides (–Br and –Cl) or electron-withdrawing residues (–NO_2_, –CN, and –CO_2_CH_3_) attached to the phenyl ring increased the electrophilicity and, subsequently, the antimicrobial activity of CNMA derivatives [89]. Furthermore, 4-BromoCNMA, 4-ChloroCNMA, and 4-NitroCNMA have their substitutions on the fourth (*para*) position, indicating the crucial role of the steric effect in the efficacy displayed. This is corroborated by a previous report that the antimicrobial activity of CNMA analogs—methoxycinnamaldehyde, nitrocinnamaldehyde and bromo-substituted CNMA—decreased by movement of substituents from the *para-* to the *ortho-* position [3,89]. The weak activity at the *ortho*-position was attributed to the ability of the steric bulk of substituents to block the electrophilic β-carbon site and prevent nucleophilic attack and impede reactivity of the derivatives [89]. In this study, 4-BromoCNMA, 4-ChloroCNMA, and 4-NitroCNMA exhibited strong antipathogenic effects on *V. parahaemolyticus*, whereas CNMA had weak inhibitory effects. This suggests that halogens and nitro group substituents on the CNMA moiety enhanced their inhibitory properties.

## 5. Conclusions

Seafood products and their processing facilities are prone to contamination by biofilm-forming bacteria such as *V. parahaemolyticus* at various stages, with the inherent risk of foodborne illnesses. In this study, CNMA derivatives—4-BromoCNMA, 4-ChloroCNMA, and 4-NitroCNMA—exhibited antimicrobial properties against *V. parahaemolyticus* by suppressing its numerous virulence factors, including in vitro and biotic surface biofilms, motility, adhesive factors, protease secretion, indole production, and virulence-related transcriptomic changes. Therefore, this study identified CNMA derivatives as potential broad-spectrum antimicrobial agents to treat biofilm-mediated *Vibrio* infections, to use for surface disinfection in food processing industries to limit the activities of foodborne pathogens, and to enhance the shelf life of seafood.

## Data Availability

The authors confirm that the data supporting the findings of this study are available within the article and/or its Supplementary Materials.

## Supplementary Materials

The following are available online at https://www.mdpi.com/article/10.3390/pharmaceutics13122176/s1, Table S1: Minimum Inhibitory Concentration (MICs) of Cinnamaldehyde and its Derivatives against *V. parahaemolyticus* and *V. harveyi*. Table S2: Primer sequences for qRT-PCR. Figure S1: Antibiofilm activity of CNMA and its derivatives (A) and biofilm dispersing effects of CNMA and its derivatives against *V. parahaemolyticus* (B).

## References

[1] Drake S.L., DePaola A., Jaykus L.A. (2007). An overview of *Vibrio vulnificus* and *Vibrio parahaemolyticus*. Compr. Rev. Food Sci. Food Saf..

[2] Huang J.Y., Henao O.L., Griffin P.M., Vugia D.J., Cronquist A.B., Hurd S., Tobin-D’Angelo M., Ryan P., Smith K., Lathrop S. (2016). Infection with pathogens transmitted commonly through food and the effect of increasing use of culture-independent diagnostic tests on surveillance—Foodborne diseases active surveillance network, 10 US sites, 2012–2015. Morb. Mortal. Wkly. Rep..

[3] Brackman G., Defoirdt T., Miyamoto C., Bossier P., Van Calenbergh S., Nelis H., Coenye T. (2008). Cinnamaldehyde and cinnamaldehyde derivatives reduce virulence in *Vibrio* spp. by decreasing the DNA-binding activity of the quorum sensing response regulator LuxR. BMC Microbiol..

[4] Baker-Austin C., Oliver J.D., Alam M., Ali A., Waldor M.K., Qadri F., Martinez-Urtaza J. (2018). *Vibrio* spp. infections. Nat. Rev. Dis. Prim..

[5] Khouadja S., Lamari F., Bakhrouf A. (2013). Characterization of *Vibrio parahaemolyticus* isolated from farmed sea bass (*Dicentrarchus labrax*) during disease outbreaks. Int. Aquat. Res..

[6] Rasko D.A., Sperandio V. (2010). Anti-virulence strategies to combat bacteria-mediated disease. Nat. Rev. Drug Discov..

[7] Ashrafudoulla M., Mizan M., Rahaman F., Park H., Byun K.-H., Lee N., Park S.H., Ha S.-D. (2019). Genetic relationship, virulence factors, drug resistance profile and biofilm formation ability of *Vibrio parahaemolyticus* isolated from mussel. Front. Microbiol..

[8] Zhang Y., Qiu Y., Tan Y., Guo Z., Yang R., Zhou D. (2012). Transcriptional regulation of *opaR, qrr2–4* and *aphA* by the master quorum-sensing regulator OpaR in *Vibrio parahaemolyticus*. PLoS ONE.

[9] Lu R., Osei-Adjei G., Huang X., Zhang Y. (2018). Role and regulation of the orphan AphA protein of quorum sensing in pathogenic *Vibrios*. Future Microbiol..

[10] Lu R., Tang H., Qiu Y., Yang W., Yang H., Zhou D., Huang X., Hu L., Zhang Y. (2019). Quorum sensing regulates the transcription of lateral flagellar genes in *Vibrio parahaemolyticus*. Future Microbiol..

[11] Yuan L., Hansen M.F., Røder H.L., Wang N., Burmølle M., He G. (2020). Mixed-species biofilms in the food industry: Current knowledge and novel control strategies. Crit. Rev. Food Sci. Nutr..

[12] Mizan M.F.R., Ashrafudoulla M., Sadekuzzaman M., Kang I., Ha S.-D. (2018). Effects of NaCl, glucose, and their combinations on biofilm formation on black tiger shrimp (*Penaeus monodon*) surfaces by *Vibrio parahaemolyticus*. Food Control.

[13] Han N., Mizan M.F.R., Jahid I.K., Ha S.-D. (2016). Biofilm formation by *Vibrio parahaemolyticus* on food and food contact surfaces increases with rise in temperature. Food Control.

[14] Concha-Meyer A., Schöbitz R., Brito C., Fuentes R. (2011). Lactic acid bacteria in an alginate film inhibit *Listeria monocytogenes* growth on smoked salmon. Food Control.

[15] Elexson N., Yaya R., Nor A.M., Kantilal H.K., Ubong A., Nishibuchi M., Yoshitsugu N., Son R. (2014). Biofilm assessment of *Vibrio parahaemolyticus* from seafood using random amplified polymorphism DNA-PCR. Int. Food Res. J..

[16] Xu X., Cheng J., Wu Q., Zhang J., Xie T. (2016). Prevalence, characterization, and antibiotic susceptibility of *Vibrio parahaemolyticus* isolated from retail aquatic products in North China. BMC Microbiol..

[17] Ahmed H.A., El Bayomi R.M., Hussein M.A., Khedr M.H.E., Remela E.M.A., El-Ashram A.M.M. (2018). Molecular characterization, antibiotic resistance pattern and biofilm formation of *Vibrio parahaemolyticus* and *V. cholerae* isolated from crustaceans and humans. Int. J. Food Microbiol..

[18] Cao J., Liu H., Wang Y., He X., Jiang H., Yao J., Xia F., Zhao Y., Chen X. (2021). Antimicrobial and antivirulence efficacies of citral against foodborne pathogen *Vibrio parahaemolyticus* RIMD2210633. Food Control.

[19] Firmino D.F., Cavalcante T.T.A., Gomes G.A., Firmino N., Rosa L.D., de Carvalho M.G., Catunda F.E.A. (2018). Antibacterial and antibiofilm activities of *Cinnamomum* sp. essential oil and cinnamaldehyde: Antimicrobial activities. Sci. World J..

[20] Adams T.B., Cohen S.M., Doull J., Feron V.J., Goodman J.I., Marnett L.J., Munro I.C., Portoghese P.S., Smith R.L., Waddell W.J. (2004). The FEMA GRAS assessment of cinnamyl derivatives used as flavor ingredients. Food Chem. Toxicol..

[21] Zhu L., Olsen C., McHugh T., Friedman M., Jaroni D., Ravishankar S. (2014). Apple, Carrot, and Hibiscus edible films containing the plant antimicrobials carvacrol and cinnamaldehyde inactivate *Salmonella* Newport on organic leafy greens in sealed plastic bags. J. Food Sci..

[22] Han C., Wang J., Li Y., Cui Y. (2013). *In vitro* antimicrobial activity and effect on *E. coli* integrity of cinnamon essential oil and rhubarb ethanol extract. Food Sci. Technol. Res..

[23] Shen Q., Zhou W., Hu L., Qi Y., Ning H., Chen J., Mo H. (2017). Bactericidal activity of alpha-bromocinnamaldehyde against persisters in *Escherichia coli*. PLoS ONE.

[24] Kot B., Wicha J., Piechota M., Wolska K., Gruzewska A. (2015). Antibiofilm activity of trans-cinnamaldehyde, *p*-coumaric, and ferulic acids on uropathogenic *Escherichia coli*. Turk. J. Med. Sci..

[25] Kim Y.-G., Lee J.-H., Kim S.-I., Baek K.-H., Lee J. (2015). Cinnamon bark oil and its components inhibit biofilm formation and toxin production. Int. J. Food Microbiol..

[26] Malheiro J.F., Maillard J.Y., Borges F., Simões M. (2019). Evaluation of cinnamaldehyde and cinnamic acid derivatives in microbial growth control. Int. Biodeterior. Biodegrad..

[27] Kavanaugh N.L., Ribbeck K. (2012). Selected antimicrobial essential oils eradicate *Pseudomonas* spp. and *Staphylococcus aureus* biofilms. Appl. Environ. Microbiol..

[28] Beema Shafreen R.M., Selvaraj C., Singh S.K., Karutha Pandian S. (2014). *In silico* and *in vitro* studies of cinnamaldehyde and their derivatives against LuxS in *Streptococcus pyogenes*: Effects on biofilm and virulence genes. J. Mol. Recognit..

[29] Da Nóbrega Alves D., Monteiro A.F.M., Andrade P.N., Lazarini J.G., Abílio G.M.F., Guerra F.Q.S., Scotti M.T., Scotti L., Rosalen P.L., Castro R.D. (2020). Docking prediction, antifungal activity, anti-biofilm effects on *Candida* spp., and toxicity against human cells of cinnamaldehyde. Molecules.

[30] Sun Q., Shang B., Wang L., Lu Z., Liu Y. (2016). Cinnamaldehyde inhibits fungal growth and aflatoxin B1 biosynthesis by modulating the oxidative stress response of *Aspergillus flavus*. Appl. Microbiol. Biotechnol..

[31] Banu S.F., Rubini D., Murugan R., Vadivel V., Gowrishankar S., Pandian S.K., Nithyanand P. (2018). Exploring the antivirulent and sea food preservation efficacy of essential oil combined with DNase on *Vibrio parahaemolyticus*. LWT.

[32] Lu C., Liu H., Shangguan W., Chen S., Zhong Q. (2021). Antibiofilm activities of the cinnamon extract against *Vibrio parahaemolyticus* and *Escherichia coli*. Arch. Microbiol..

[33] Zheng X., Feyaerts A.F., Van Dijck P., Bossier P. (2020). Inhibitory activity of essential oils against *Vibrio campbellii* and *Vibrio parahaemolyticus*. Microorganisms.

[34] Doyle A.A., Stephens J.C. (2019). A review of cinnamaldehyde and its derivatives as antibacterial agents. Fitoterapia.

[35] Khadke S.K., Lee J.-H., Woo J.-T., Lee J. (2019). Inhibitory effects of honokiol and magnolol on biofilm formation by *Acinetobacter baumannii*. Biotechnol. Bioprocess Eng..

[36] Seo S., Jung J., Kim C.Y., Kang H., Lee I.H. (2021). Antimicrobial peptides encounter resistance of aureolysin during their action on *Staphylococcus aureus* biofilm. Biotechnol. Bioprocess Eng..

[37] Lee J.-H., Kim Y.-G., Raorane C.J., Ryu S.Y., Shim J.-J., Lee J. (2019). The anti-biofilm and anti-virulence activities of trans-resveratrol and oxyresveratrol against uropathogenic *Escherichia coli*. Biofouling.

[38] Kim Y.-G., Lee J.-H., Lee S., Lee Y.-K., Hwang B.S., Lee J. (2021). Antibiofilm activity of phorbaketals from the marine sponge *Phorbas* sp. against *Staphylococcus aureus*. Mar. Drugs.

[39] Heydorn A., Nielsen A.T., Hentzer M., Sternberg C., Givskov M., Ersbøll B.K., Molin S. (2000). Quantification of biofilm structures by the novel computer program COMSTAT. Microbiology.

[40] Raorane C.J., Lee J.-H., Lee J. (2020). Rapid killing and biofilm inhibition of multidrug-resistant *Acinetobacter baumannii* strains and other microbes by iodoindoles. Biomolecules.

[41] Heering J., Alvarado A., Ringgaard S. (2017). Induction of cellular differentiation and single cell imaging of *Vibrio parahaemolyticus* swimmer and swarmer cells. J. Vis. Exp..

[42] Sathiyamoorthi E., Faleye O.S., Lee J.-H., Raj V., Lee J. (2021). Antibacterial and antibiofilm activities of chloroindoles against *Vibrio parahaemolyticus*. Front. Microbiol..

[43] Sethupathy S., Sathiyamoorthi E., Kim Y.-G., Lee J.-H., Lee J. (2020). Antibiofilm and antivirulence properties of indoles against *Serratia marcescens*. Front. Microbiol..

[44] Mizan M.F.R., Jahid I.K., Kim M., Lee K.-H., Kim T.J., Ha S.-D. (2016). Variability in biofilm formation correlates with hydrophobicity and quorum sensing among *Vibrio parahaemolyticus* isolates from food contact surfaces and the distribution of the genes involved in biofilm formation. Biofouling.

[45] He Z., Huang Z., Jiang W., Zhou W. (2019). Antimicrobial activity of cinnamaldehyde on *Streptococcus mutans* biofilms. Front. Microbiol..

[46] Di Martino P., Fursy R., Bret L., Sundararaju B., Phillips R.S. (2003). Indole can act as an extracellular signal to regulate biofilm formation of *Escherichia coli* and other indole-producing bacteria. Can. J. Microbiol..

[47] Mueller R.S., Beyhan S., Saini S.G., Yildiz F.H., Bartlett D.H. (2009). Indole acts as an extracellular cue regulating gene expression in *Vibrio cholerae*. J. Bacteriol..

[48] Han T.H., Lee J.-H., Cho M.H., Wood T.K., Lee J. (2011). Environmental factors affecting indole production in *Escherichia coli*. Res. Microbiol..

[49] Toushik S.H., Kim K., Ashrafudoulla M., Mizan M.F.R., Roy P.K., Nahar S., Kim Y., Ha S.-D. (2021). Korean kimchi-derived lactic acid bacteria inhibit foodborne pathogenic biofilm growth on seafood and food processing surface materials. Food Control.

[50] Kim Y.-G., Lee J.-H., Gwon G., Kim S.-I., Park J.G., Lee J. (2016). Essential oils and eugenols inhibit biofilm formation and the virulence of *Escherichia coli* O157: H7. Sci. Rep..

[51] Li X.-H., Lee J.-H. (2017). Antibiofilm agents: A new perspective for antimicrobial strategy. J. Microbiol..

[52] Sharma A., Bajpai V.K., Baek K.H. (2013). Determination of antibacterial mode of action of *Allium sativum* essential oil against foodborne pathogens using membrane permeability and surface characteristic parameters. J. Food Saf..

[53] Denis K., Le Bris M., Le Guennec L., Barnier J.-P., Faure C., Gouge A., Bouzinba-Ségard H., Jamet A., Euphrasie D., Durel B. (2019). Targeting Type IV pili as an antivirulence strategy against invasive meningococcal disease. Nat. Microbiol..

[54] McCarter L.L. (2004). Dual flagellar systems enable motility under different circumstances. J. Mol. Microbiol. Biotechnol..

[55] Van Houdt R., Michiels C.W. (2010). Biofilm formation and the food industry, a focus on the bacterial outer surface. J. Appl. Microbiol..

[56] Paranjpye R.N., Johnson A.B., Baxter A.E., Strom M.S. (2007). Role of type IV pilins in persistence of *Vibrio vulnificus* in *Crassostrea virginica* oysters. Appl. Environ. Microbiol..

[57] Letchumanan V., Chan K.-G., Lee L.-H. (2014). *Vibrio parahaemolyticus*: A review on the pathogenesis, prevalence, and advance molecular identification techniques. Front. Microbiol..

[58] Ottaviani D., Santarelli S., Bacchiocchi S., Masini L., Ghittino C., Bacchiocchi I. (2005). Presence of pathogenic *Vibrio parahaemolyticus* strains in mussels from the Adriatic Sea, Italy. Food Microbiol..

[59] Lee J.-H., Wood T.K., Lee J. (2015). Roles of indole as an interspecies and interkingdom signaling molecule. Trends Microbiol..

[60] Wyeth F.J.S. (1919). The effects of acids, alkalies, and sugars on the growth and indole formation of *Bacillus coli*: A report to the medical research committee. Biochem. J..

[61] Yohannes E., Barnhart D.M., Slonczewski J.L. (2004). pH-dependent catabolic protein expression during anaerobic growth of *Escherichia coli* K-12. J. Bacteriol..

[62] Lee J.-H., Kim Y.-G., Kim C.-J., Lee J.-C., Cho M.H., Lee J. (2012). Indole-3-acetaldehyde from *Rhodococcus* sp. BFI 332 inhibits *Escherichia coli* O157: H7 biofilm formation. Appl. Microbiol. Biotechnol..

[63] Malheiro J., Gomes I., Borges A., Bastos M., Maillard J.Y., Borges F., Simões M. (2016). Phytochemical profiling as a solution to palliate disinfectant limitations. Biofouling.

[64] Niu C., Gilbert E.S. (2004). Colorimetric method for identifying plant essential oil components that affect biofilm formation and structure. Appl. Environ. Microbiol..

[65] Alkawash M.A., Soothill J.S., Schiller N.L. (2006). Alginate lyase enhances antibiotic killing of mucoid *Pseudomonas aeruginosa* in biofilms. APMIS.

[66] Darouiche R.O., Mansouri M.D., Gawande P.V., Madhyastha S. (2009). Antimicrobial and antibiofilm efficacy of triclosan and DispersinB^®^ combination. J. Antimicrob. Chemother..

[67] Friedman M. (2017). Chemistry, antimicrobial mechanisms, and antibiotic activities of cinnamaldehyde against pathogenic bacteria in animal feeds and human foods. J. Agric. Food Chem..

[68] Wolfram J., Zhu M., Yang Y., Shen J., Gentile E., Paolino D., Fresta M., Nie G., Chen C., Shen H. (2015). Safety of nanoparticles in medicine. Curr. Drug Targets.

[69] Zhu Y., Li C., Cui H., Lin L. (2021). Encapsulation strategies to enhance the antibacterial properties of essential oils in food system. Food Control.

[70] Zhan Q., Xu Y., Zhan L., Wang B., Guo Y., Wu X., Ai W., Song Z., Yu F. (2021). Chromone derivatives CM3a potently eradicate *Staphylococcus aureus* biofilms by inhibiting cell adherence. Infect. Drug Resist..

[71] Donlan R.M. (2002). Biofilms: Microbial life on surfaces. Emerg. Infect. Dis..

[72] Lee C.-Y., Cheng M.-F., Yu M.-S., Pan M.-J. (2002). Purification and characterization of a putative virulence factor, serine protease, from *Vibrio parahaemolyticus*. FEMS Microbiol. Lett..

[73] Valiente E., Lee C.T., Hor L.I., Fouz B., Amaro C. (2008). Role of the metalloprotease Vvp and the virulence plasmid pR99 of *Vibrio vulnificus* serovar E in surface colonization and fish virulence. Environ. Microbiol..

[74] Denkin S.M., Nelson D.R. (2004). Regulation of *Vibrio anguillarum* empA metalloprotease expression and its role in virulence. Appl. Environ. Microbiol..

[75] Miyoshi S. (2006). *Vibrio vulnificus* infection and metalloprotease. J. Dermatol..

[76] Maurer L.M., Yohannes E., Bondurant S.S., Radmacher M., Slonczewski J.L. (2005). pH regulates genes for flagellar motility, catabolism, and oxidative stress in *Escherichia coli* K-12. J. Bacteriol. Res..

[77] Blankenhorn D., Phillips J., Slonczewski J.L. (1999). Acid-and base-induced proteins during aerobic and anaerobic growth of *Escherichia coli* revealed by two-dimensional gel electrophoresis. J. Bacteriol. Res..

[78] Lee J., Jayaraman A., Wood T.K. (2007). Indole is an inter-species biofilm signal mediated by SdiA. BMC Microbiol..

[79] Wang L., Ling Y., Jiang H., Qiu Y., Qiu J., Chen H., Yang R., Zhou D. (2013). AphA is required for biofilm formation, motility, and virulence in pandemic *Vibrio parahaemolyticus*. Int. J. Food Microbiol..

[80] Qian H., Li W., Guo L., Tan L., Liu H., Wang J., Pan Y., Zhao Y. (2020). Stress response of *Vibrio parahaemolyticus* and *Listeria monocytogenes* biofilms to different modified atmospheres. Front. Microbiol..

[81] Zhou D., Yan X., Qu F., Wang L., Zhang Y., Hou J., Hu Y., Li J., Xin S., Qiu J. (2013). Quorum sensing modulates transcription of cpsQ-mfpABC and mfpABC in *Vibrio parahaemolyticus*. Int. J. Food Microbiol..

[82] Lee J., Bansal T., Jayaraman A., Bentley W.E., Wood T.K. (2007). Enterohemorrhagic *Escherichia coli* biofilms are inhibited by 7-hydroxyindole and stimulated by isatin. Appl. Environ. Microbiol..

[83] Sun X.-H., Hao L.-R., Xie Q.-C., Lan W.-Q., Zhao Y., Pan Y.-J., Wu V.C.H. (2020). Antimicrobial effects and membrane damage mechanism of blueberry (*Vaccinium corymbosum* L.) extract against *Vibrio parahaemolyticus*. Food Control.

[84] Lowery C.A., Dickerson T.J., Janda K.D. (2008). Interspecies and interkingdom communication mediated by bacterial quorum sensing. Chem. Soc. Rev..

[85] Pompeani A.J., Irgon J.J., Berger M.F., Bulyk M.L., Wingreen N.S., Bassler B.L. (2008). The *Vibrio harveyi* master quorum-sensing regulator, LuxR, a TetR-type protein is both an activator and a repressor: DNA recognition and binding specificity at target promoters. Mol. Microbiol..

[86] Zhao J., Chen M., Quan C.S., Fan S.D. (2015). Mechanisms of quorum sensing and strategies for quorum sensing disruption in aquaculture pathogens. J. Fish Dis..

[87] Niu C., Afre S., Gilbert E.S. (2006). Subinhibitory concentrations of cinnamaldehyde interfere with quorum sensing. Lett. Appl. Microbiol..

[88] Shaikh M.H., Subhedar D.D., Shingate B.B., Khan F.A.K., Sangshetti J.N., Khedkar V.M., Nawale L., Sarkar D., Navale G.R., Shinde S.S. (2016). Synthesis, biological evaluation and molecular docking of novel coumarin incorporated triazoles as antitubercular, antioxidant and antimicrobial agents. Med. Chem. Res..

[89] Doyle A.A., Krämer T., Kavanagh K., Stephens J.C. (2019). Cinnamaldehydes: Synthesis, antibacterial evaluation, and the effect of molecular structure on antibacterial activity. Results Chem..

