# Supplementary material for: Inhibitory Effects of Cinnamaldehyde Derivatives on Biofilm Formation and Virulence Factors in Vibrio Species
Source: Pharmaceutics. 2021 Dec 17;13(12):2176. doi: 10.3390/pharmaceutics13122176 (PMC8708114; doi:10.3390/pharmaceutics13122176)
Supplement: Supplementary file 1 [file pharmaceutics-13-02176-s001.zip › pharmaceutics-1490508-supplementary.pdf]

pharmaceutics

MDPI

Supplementary Information

Inhibitory Effects of Cinnamaldehyde Derivatives on Biofilm Formation and Virulence Factors in *Vibrio* Species

Olajide Sunday Faleye †, Ezhaveni Sathiyamoorthi †, Jin-Hyung Lee * and Jintae Lee *

Table S1. Minimum Inhibitory Concentration (MICs) of Cinnamaldehyde and its Derivatives Against *V. parahaemolyticus* and *V. harveyi*.

Chemical Name	Structure	*V. parahaemolyticus*	*V. harveyi*
4-DimethylaminoCNMA	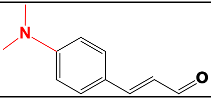	>500	>500
4-FluoroCNMA	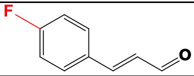	175	175
2-NitroCNMA	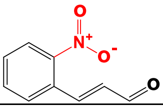	250	300
2-MethoxyCNMA	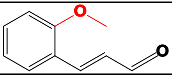	175	175
**4-ChloroCNMA**	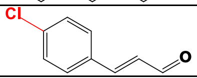	50	50
CNMAoxime	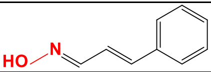	>500	>500
**4-NitroCNMA**	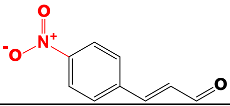	50	50
4-MethoxyCNMA	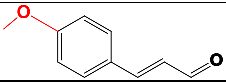	300	275
**4-BromoCNMA**	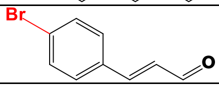	50	50
α-MethylCNMA	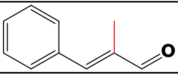	>500	>500
**CNMA**	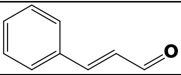	200	200

Table S2. Primer sequences for qRT-PCR.

Genes	Functions	Primer Sequences
aphA	Acid phosphatase AphA; Biofilm, motility	5′-ACACCCAACCGTTCGTGATG-3′<br>5′-GTTGAAGGCGTTGCGTAGTAAG-3′
cpsA	EPS production genes	5′-GCGCACAACGAAGAATATCG-3′<br>5′--3′CCATCTTATCGAGCGTGTCG
luxI	QS regulator	5′-GTG GAT GCT GGC GTT TAT TAC -3′<br>5′-TTT GGG AGC ACT CTG TTG AC -3′
luxS	Autoinducer binding domain-containing protein; QS	5′-GAT GGG ATG TCG CAC TGG TTT-3′<br>5′- ACT TGC TGT TCA GAA GGC GTA-3′
mshA	Type IV pilin MshA	5′-GGTTTCGTTTAGGTCACG -3′<br>5′-CGTCGAAATGTCGGCGG-3′
opaR	Transcriptional regulator OpaR; QS	5′-TGTCTACCAACCGCACTAACC-3′<br>5′-GCTCTTTCAACTCGGCTTCAC-3′
oxyR	DNA-binding transcriptional regulator OxyR; regulator of growth, biofilm formation and motility	5′-TCG TCA GCT AGA GGA AGG-3′<br>5′-TGG TCG CGT AAG CAA TGC-3′
tnaA	Tryptophanase	5′-TGA AGA AGT TGG TCC GAA TAA CGT G-3′<br>5′-CTT TGT ATT CTG CTT CAC GCT GCT T-3′
qsvR	~~an~~ AraC-type transcriptional regulator QsvR; QS regulator to control virulence	5′-TAC ACC GCC ACC CAT AAC G-3′<br>5′-AGC CAT TCT CGC CAG GTA TG-3′
fliA	Motility/Chemotaxis	5′-TAAGCGTATTGCTCACCACCT-3′<br>5′-GCTCGCACCTTTAGAACCAT-3′
tdh	Thermostable direct hemolysin (TDH)	5′-GTAAAGGTCTCTGACTTTTGGAC-3′<br>5′-TGGAATAGAACCTTCATCTTCACC-3′
vopS	T3SS effector adenosine monophosphate-protein transferase VopS; virulence genes (secretion systems)	5′-AAGGTAGGGCAACGCAAAGA-3′<br>5′-AGCAGCACGACAGCAATACT-3′
vmrA	Sodium-coupled multidrug efflux MATE transporter VmrA	5′-GGTGTTGTGTTCGTGGTATTG-3′<br>5′-CTTGGATGCTCGGTTCTACTG-3′
vmeB	Multidrug efflux RND transporter permease subunit VmeB	5′-CTGCGACCATTACACTGACTT-3′<br>5′-GTGTGTAAAGTCTGGATCGTC-3′
ef-Tu	Elongation factor Tu	5′-CACTAAACGGCGAAGAGCA-3′<br>5′-CCACGACCTTGGATTGAGAA-3′
fadL	Long-chain fatty acid outer membrane channel/bacteriophage T2 receptor	5′-ACGATAAAGGTCAGGAAATCAC-3′<br>5′-GTATTGGATGCTGTAATGTACGG-3′
nusA	Transcription termination factor NusA	5′-TGTTTATCACTCGTTCTAAGCCT-3′<br>5′-GTTTGTCATTTGTTTTCACTGCG-3′
16S rRNA	Housekeeping gene	5′-TATCCTTGTTTGCCAGCGAG-3′<br>5′-CTACGACGCACTTTTTGGGA-3′

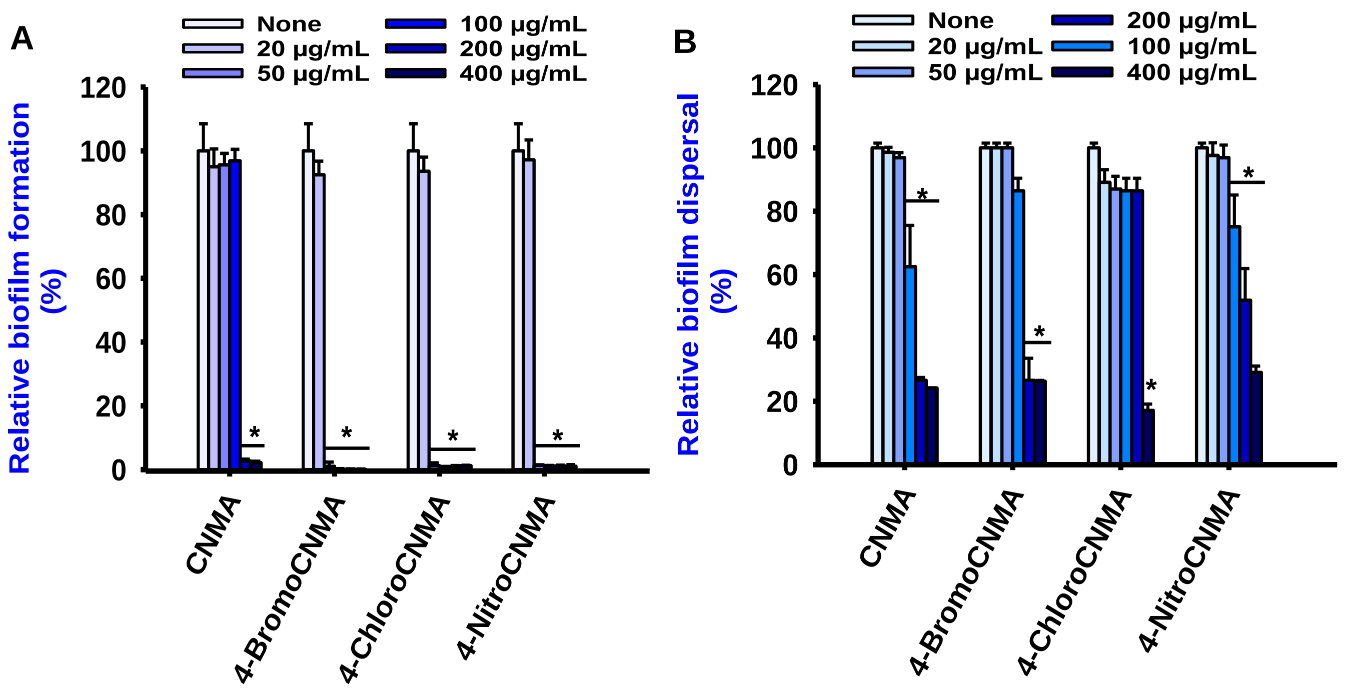

Figure S1. Antibiofilm activity of CNMA and its derivatives (**A**) and biofilm dispersing effects of CNMA and its derivatives against *V.parahaemolyticus* (**B**), * denotes a significant difference at $p < 0.05$.
